# Supplementary material for: The short-chain fatty acid pentanoate suppresses autoimmunity by modulating the metabolic-epigenetic crosstalk in lymphocytes
Source: Nat Commun. 2019 Feb 15;10:760. doi: 10.1038/s41467-019-08711-2 (PMC6377655; doi:10.1038/s41467-019-08711-2)
Supplement: Supplementary file 1 — Supplementary Information [file 41467_2019_8711_MOESM1_ESM.pdf]

Supplementary Information

The short-chain fatty acid pentanoate suppresses autoimmunity by modulating the metabolic-epigenetic crosstalk in lymphocytes

Luu et al.

Supplementary Figures

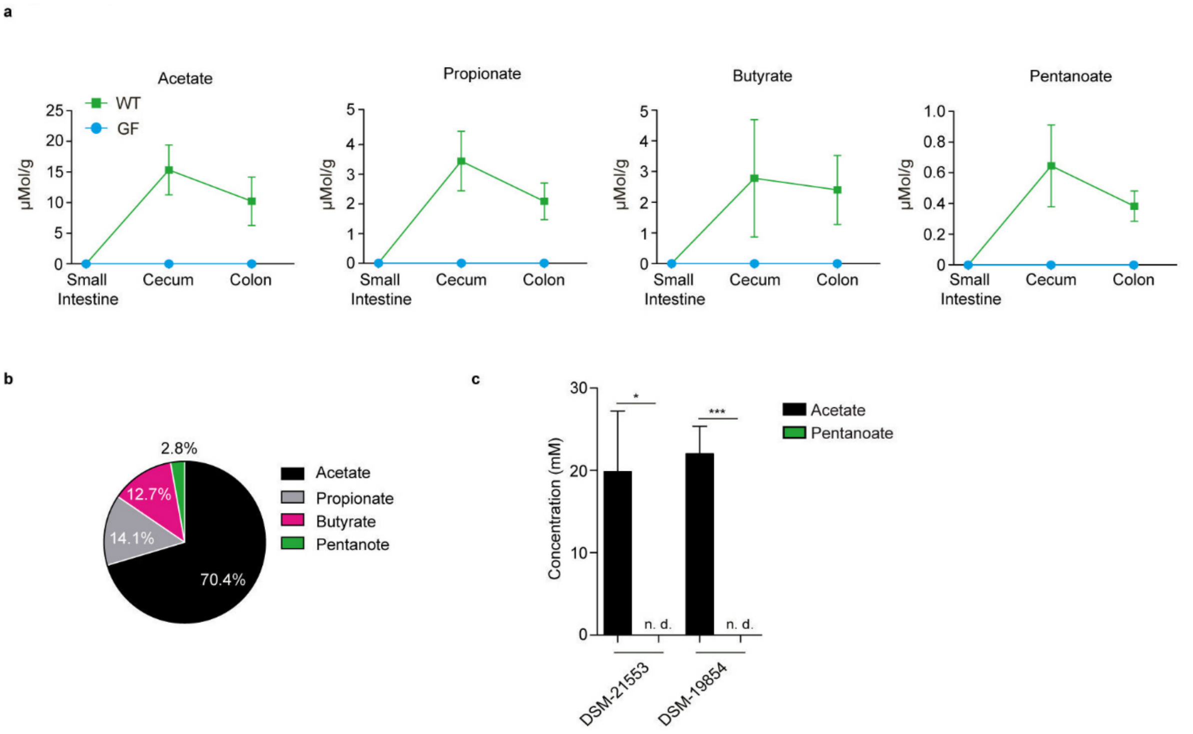

Supplementary Fig. 1 Microbiota-dependent production of pentanoate. **a** Concentrations of SCFAs in fecal extracts of WT (green squares) and GF (blue circles) mice were determined by UHPLC-MS (n = 4 mice per group). **b** Composition of the SCFAs (acetate, propionate butyrate, and pentanoate) in the cecum of WT mice. **c** Human isolates *Prevotella histicola* (DSM-19854 and DSM-21553) were grown in thioglycollate medium for 5 days. Culture supernatants were analyzed by GC-MS for SCFA production. Error bars indicate SEM, n. d. = not detectable, *$P < 0.05$, ***$P < 0.001$ (Student´s *t*-test).

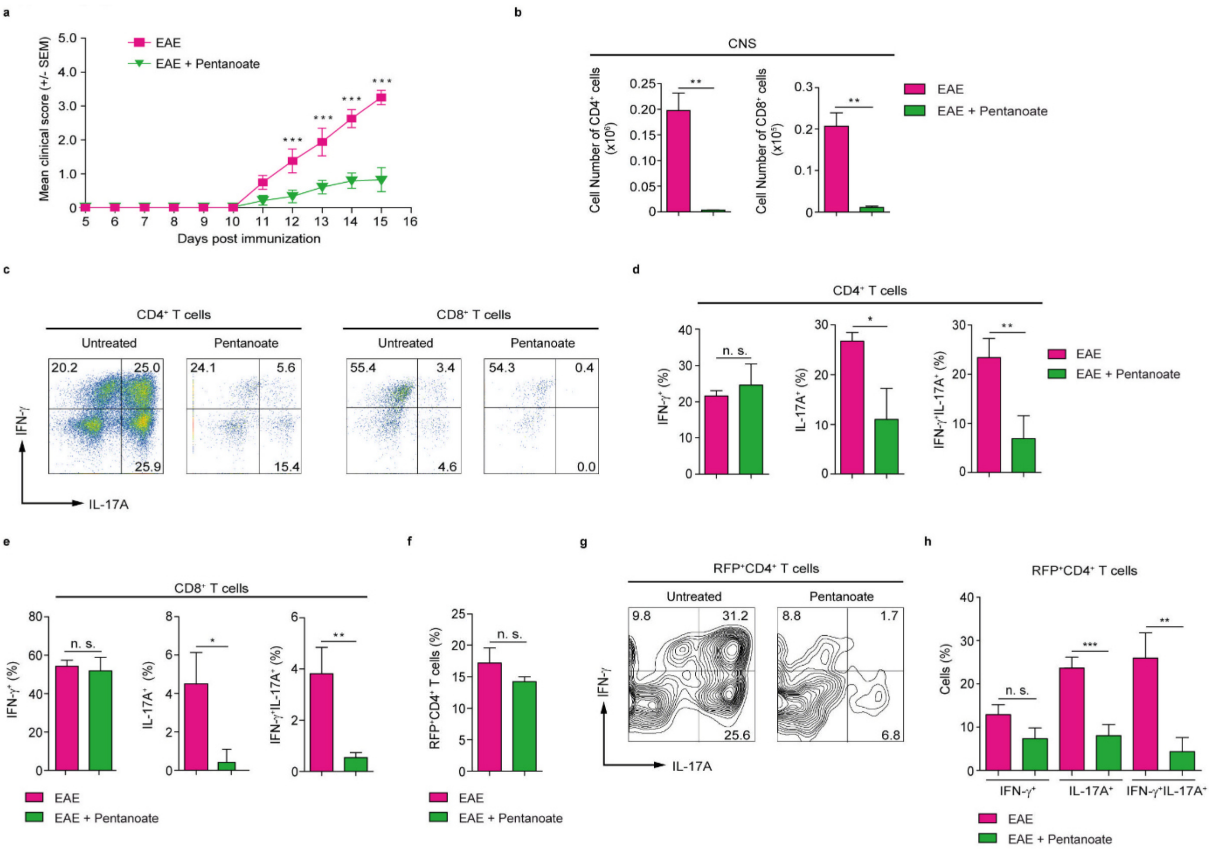

Supplementary Fig. 2 Pentanoate ameliorates progression of EAE. **a** For induction of EAE, *FIR* × *tiger* mice were immunized with MOG. Mice were treated daily with 800 mg pentanoate/kg mouse or left untreated. Two independent experiments were performed (n= 8 mice per group). Mice were monitored daily for EAE progression. The graph represents the EAE development in the presence or absence of pentanoate. **b** Total cell numbers of T lymphocytes in the inflamed CNS after pentanoate treatment. **c-e** Dot plots (**c**) and bar graphs (**d** and **e**) show the frequencies of IFN-γ^+, IL-17A$^+$ and IFN-γ^+IL-17A$^+$ cells in the inflamed CNS among CD4$^+$ and CD8$^+$ T lymphocytes, respectively. **f** The frequency of Foxp3$^+$CD4$^+$ Tregs in the CNS of mice treated with pentanoate or left untreated. **g, h** Representative contour plots (**g**) and bar graphs (**h**) indicating the frequencies of IFN-γ^+, IL-17A$^+$ and IFN-γ^+IL-17A$^+$ cells among RFP$^+$ Tregs in the CNS after treatment with pentanoate. Error bars indicate SEM. Data are analyzed by one-way ANOVA test. n. s. = not significant, **P* < 0.05, ***P* < 0.01, ****P* <0.001.

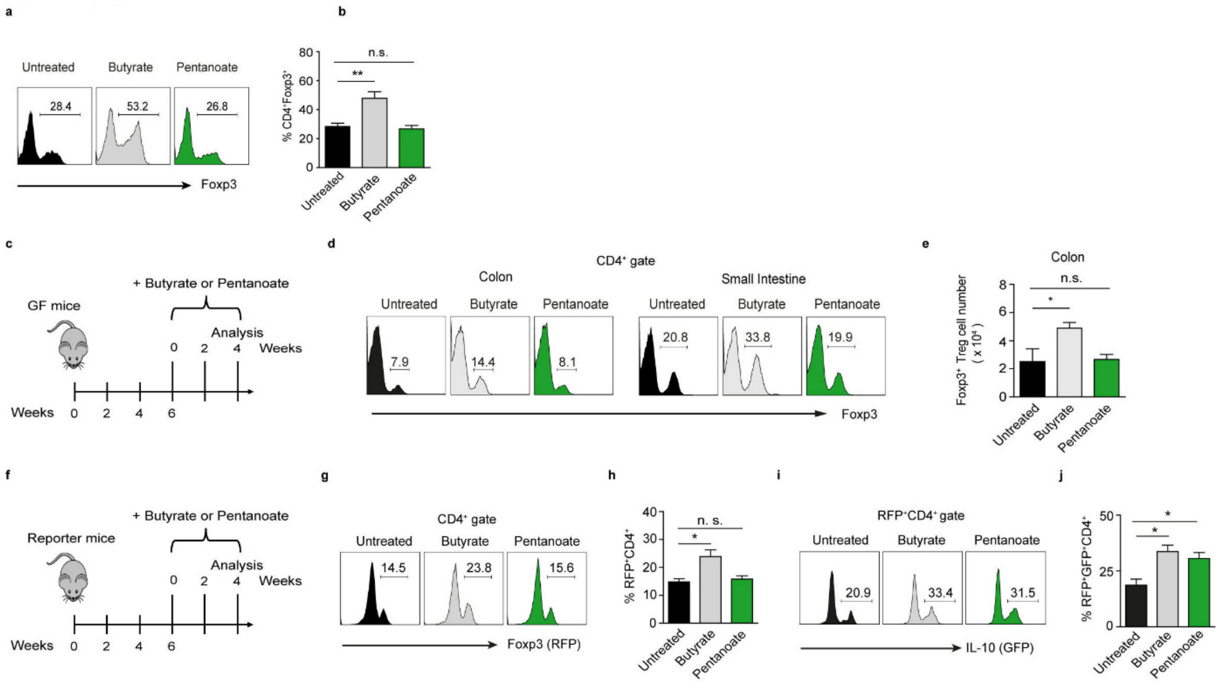

Supplementary Fig. 3 Pentanoate does not promote expansion of Tregs. **a**, **b** Flow cytometry analysis of Tregs treated with butyrate or pentanoate (1 mM) for three days. Histograms (**a**) and bar graphs (**b**) show the frequencies of Foxp3$^+$CD4$^+$ T cells. Three independent experiments were performed. **c-e** GF mice were treated orally for 4 weeks with either butyrate or pentanoate (150 mM, n =8 mice per group). Scheme of oral treatment is shown in (**c**). Dot plots (**d**) display frequencies of intestinal FoxP3$^+$CD4$^+$ T cells. Bar graphs (**d**) represent the total cell numbers of colonic Tregs after oral treatment. **f-j** Oral treatment of *FIR* × *tiger* reporter mice with SCFAs (n =8 mice per group). Scheme of oral treatment of animals with 150 mM butyrate or pentanoate is shown in (**f**). FACS analysis displaying the frequency of Foxp3$^+$CD4$^+$ T cells in colonic Tregs after SCFA treatment is shown in (**g**) and (**h**). The frequency of IL-10$^+$ cells within colonic Treg cell population after four weeks of SCFA treatment is shown (**i**) and (**j**). Two independent experiments were performed. Error bars indicate SEM, n.s. = not significant, *P < 0.05, **P < 0.01 (one-way ANOVA test).

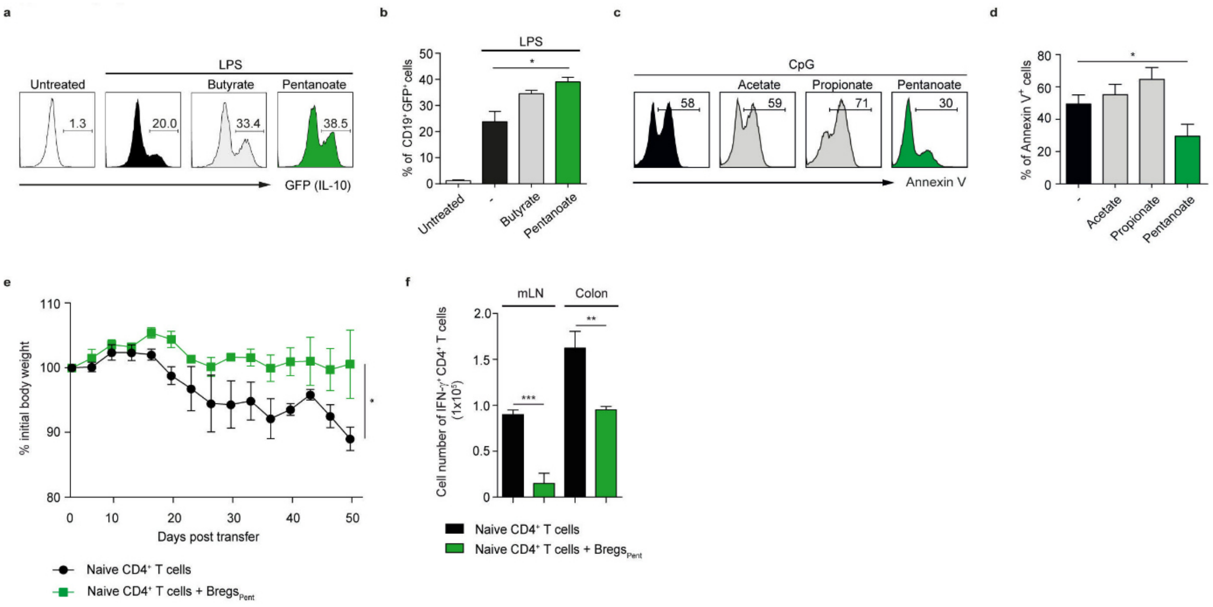

Supplementary Fig. 4 Pentanoate enhances IL-10 expression in Bregs and protects from T cell-mediated colitis. **a**, **b** B cells, isolated from spleens of *FIR* × *tiger* mice, were activated with 1 µg/ml LPS in the presence of 1 mM butyrate or 2.5 mM pentanoate for 3 days. Histograms (**a**) and bar graphs (**b**) show the frequency of IL-10^{+} cells. Three experiments were performed. **c**, **d** Bregs were generated by treating splenic B cells with CpG in the presence or absence of SCFAs (5 mM) for three days. Histograms (**c**) and bar graphs (**d**) display the frequency of Annexin V^{+} cells. Three similar experiments were performed. **e** 5x10^{5} naive CD4^{+} T cells were transferred into *Rag1*$^{-/-}$ mice to induce T cell-mediated colitis. 2 x10^{6} Bregs treated with pentanoate (Breg$_{Pent}$) were co-transferred on day 0 and 14 after T cell transfer (n = 8 mice per group). The graph represents the change of the initial body weight of recipient mice in the presence or absence of Bregs. **f** Mice were treated as described in (**e**). Cell numbers of IFN-γ^{+}CD4^{+} T cells isolated from mesenteric lymph nodes (mLN) and colon of recipient mice were counted on day 50 after colitis induction. Error bars indicate SEM, *P < 0.05, **P < 0.01, ***P <0.001 (Student´s *t*-test).

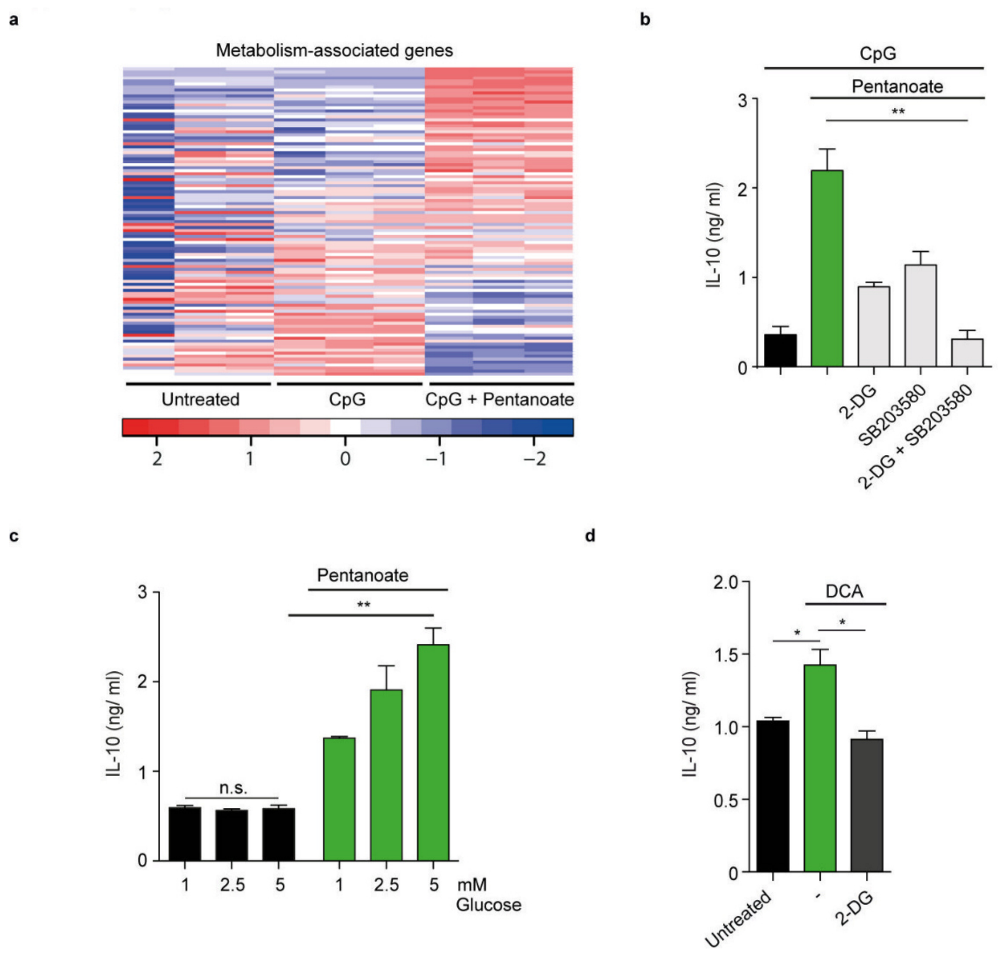

Supplementary Fig. 5 Impact of pentanoate on the metabolic state of CpG-induced Bregs. **a** Splenic B cells were stimulated with CpG and pentanoate for four days. Heatmap of metabolism-associated genes is shown (A complete list of metabolic genes was published by us previously, Shaul YD *et al.*, Cell, 2014). The FPKM values were z-transformed and plotted. **b** Bregs were generated with 4 µg/ml CpG and 5 mM pentanoate in presence of 1 mM 2-DG and 5 µM SB203580. IL-10 expression was determined on day 4 of the cell culture by flow cytometry. Three experiments were performed. **c** IL-10 secretion by Bregs treated with increasing amounts of glucose in presence or absence of pentanoate was determined by ELISA. **d** Bregs were generated with 4 µg/ml CpG and treated with DCA (5 mM) in the presence or absence of 2-DG (1 mM) for three days. IL-10 production was measured by ELISA. Error bars indicate SEM, n. s. = not significant, *$P < 0.05$, **$P < 0.01$ (Student´s *t*-test).

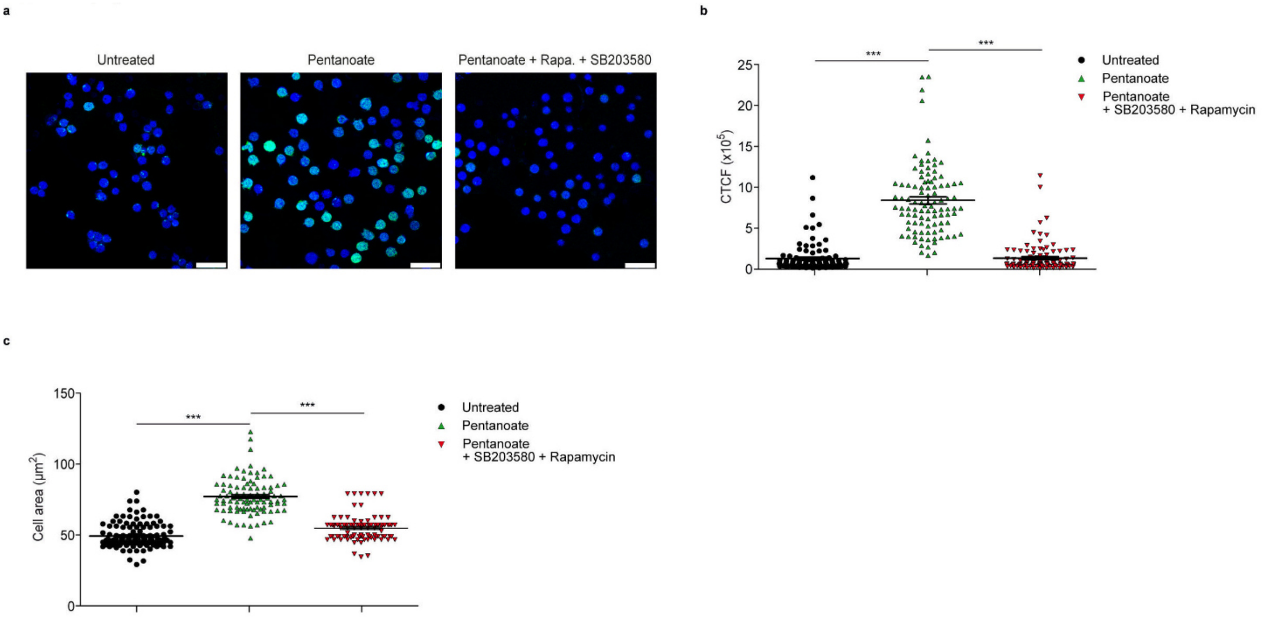

Supplementary Fig. 6 Impact of pentanoate on the cell size and IL-10 production of Bregs. **a-c** Immunofluorescence analysis of pentanoate-induced Bregs purified from spleens of *FIR* × *tiger* mice and cultured in the presence or absence of rapamycin (100 nM) and SB203580 (5 μM). IL-10^+ cells (**b**, CTCF method) and cell area (**c**) were analyzed by immunofluorescence staining (scale bar = 25 micron). CellTracker Blue CMAC Dye was used for monitoring location of B cells. ****P* <0.001 (Student´s *t*-test).

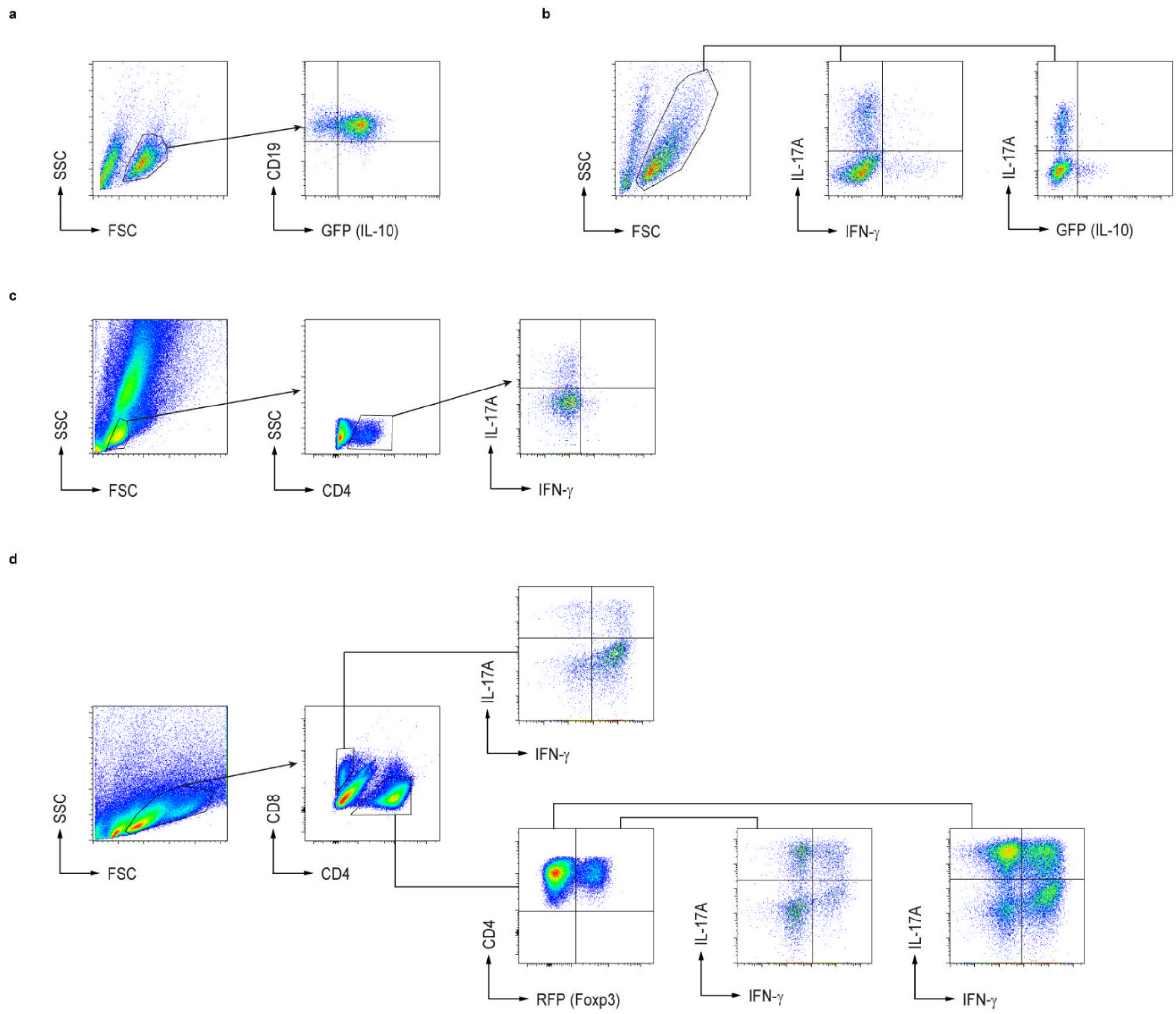

Supplementary Fig. 7 Representative gating strategy used for cell sorting and FACS analysis of T and B lymphocytes. **a** Gating strategy to sort and analyze Bregs derived from spleen of *FIR* × *tiger* mice for Fig. 4a-f, Fig. 5a-f, Fig. 6a, Fig. 6d and Fig. 6e. **b** Gating strategy for *in vitro* analysis of Th17 cells purified from spleen and LNs of WT and *FIR* × *tiger* mice (Fig. 1a, Fig. 2b and Fig. 3a). **c** Gating strategy to analyze $CD4^+$ T cells in the intestinal lamina propria (Fig. 1d and Fig. 1f). **d** Gating strategy to analyze the cell number and frequency of T lymphocytes in the CNS. T cells were analyzed for expression of IL-17A and IFN-γ (Fig. 1d-f, Fig. 6b,c and Fig. 6f,g).

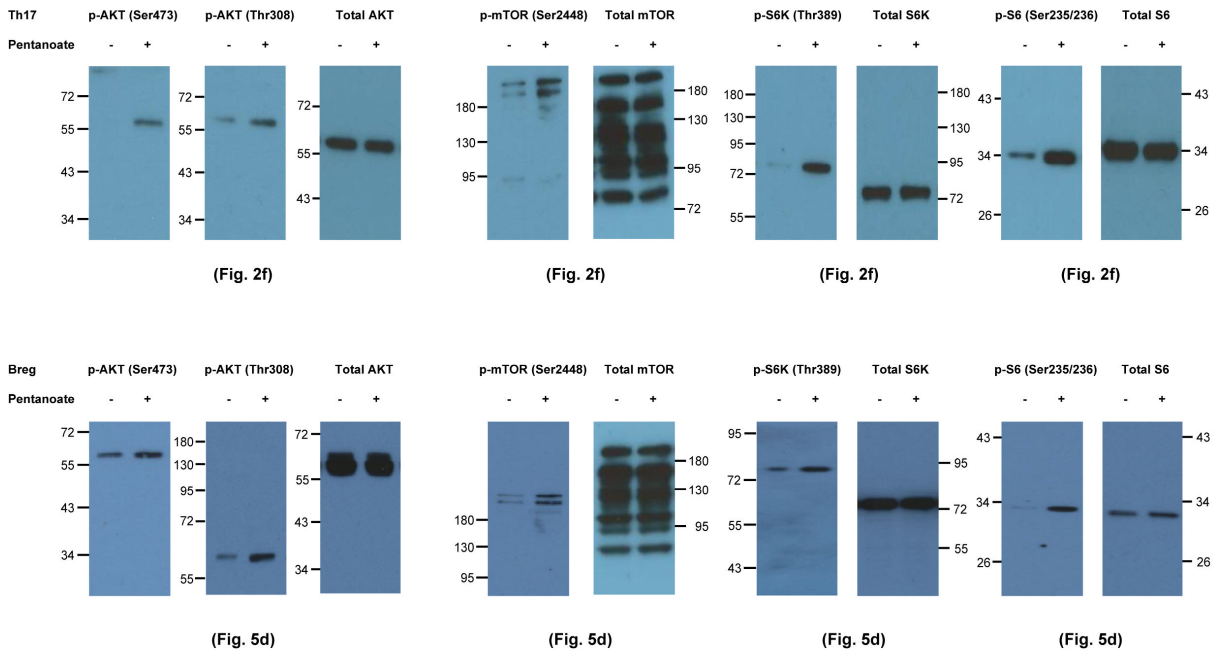

Supplementary Fig. 8 The uncropped images of western blots used to generate Fig. 2f (Th17 cells) and Fig. 5d (Bregs). All original western blots are accompanied by the localication of molecular weight.
