# Supplementary material for: The short-chain fatty acid pentanoate suppresses autoimmunity by modulating the metabolic-epigenetic crosstalk in lymphocytes
Source: Nat Commun. 2019 Feb 15;10:760. doi: 10.1038/s41467-019-08711-2 (PMC6377655; doi:10.1038/s41467-019-08711-2)
Supplement: Supplementary file 3 — Reporting Summary [file 41467_2019_8711_MOESM3_ESM.pdf]

nature research

Corresponding author(s): Alexander Visekruna

Last updated by author(s): Jan 16, 2019

Reporting Summary

Nature Research wishes to improve the reproducibility of the work that we publish. This form provides structure for consistency and transparency in reporting. For further information on Nature Research policies, see Authors & Referees and the Editorial Policy Checklist.

Statistics

For all statistical analyses, confirm that the following items are present in the figure legend, table legend, main text, or Methods section.

n/a	Confirmed	
☐	☒	The exact sample size (n) for each experimental group/condition, given as a discrete number and unit of measurement
☐	☒	A statement on whether measurements were taken from distinct samples or whether the same sample was measured repeatedly
☐	☒	The statistical test(s) used AND whether they are one- or two-sided<br>*Only common tests should be described solely by name; describe more complex techniques in the Methods section.*
☐	☒	A description of all covariates tested
☐	☒	A description of any assumptions or corrections, such as tests of normality and adjustment for multiple comparisons
☐	☒	A full description of the statistical parameters including central tendency (e.g. means) or other basic estimates (e.g. regression coefficient) AND variation (e.g. standard deviation) or associated estimates of uncertainty (e.g. confidence intervals)
☐	☒	For null hypothesis testing, the test statistic (e.g. F, t, r) with confidence intervals, effect sizes, degrees of freedom and P value noted<br>*Give P values as exact values whenever suitable.*
☒	☐	For Bayesian analysis, information on the choice of priors and Markov chain Monte Carlo settings
☒	☐	For hierarchical and complex designs, identification of the appropriate level for tests and full reporting of outcomes
☒	☐	Estimates of effect sizes (e.g. Cohen's d, Pearson's r), indicating how they were calculated

Our web collection on statistics for biologists contains articles on many of the points above.

Software and code

Policy information about availability of computer code

Data collection	FACSDiva software (version 8.1, BD Biosciences) was used to collect FACS samples.
Data analysis	FlowJo (version 10) was used to analyze flow cytometry data. GraphPad Prism 5 was used for graphs and statistical analysis. R (version 3.4.3) was used for analysis of RNA-Seq data. Agilent Seahorse Wave software was used for metabolic studies.

For manuscripts utilizing custom algorithms or software that are central to the research but not yet described in published literature, software must be made available to editors/reviewers. We strongly encourage code deposition in a community repository (e.g. GitHub). See the Nature Research guidelines for submitting code & software for further information.

Data

Policy information about availability of data

All manuscripts must include a data availability statement. This statement should provide the following information, where applicable:

- Accession codes, unique identifiers, or web links for publicly available datasets
- A list of figures that have associated raw data
- A description of any restrictions on data availability

RNA-seq data have been deposited in ArrayExpress Archive of Functional Genomics Data under the accession codes E-MTAB-6114 for Th17 cells [https://www.ebi.ac.uk/arrayexpress/experiments/E-MTAB-6114/] and E-MTAB-6115 for Bregs [https://www.ebi.ac.uk/arrayexpress/experiments/E-MTAB-6115/]. All other relevant data are available from authors upon reasonable request.

Field-specific reporting

Please select the one below that is the best fit for your research. If you are not sure, read the appropriate sections before making your selection.

☒ Life sciences ☐ Behavioural & social sciences ☐ Ecological, evolutionary & environmental sciences

For a reference copy of the document with all sections, see nature.com/documents/nr-reporting-summary-flat.pdf

Life sciences study design

All studies must disclose on these points even when the disclosure is negative.

Sample size	To determine sample size for immunological studies, power analysis (power = 80 % and significance level = 5 %) was performed. Unless otherwise indicated, animal experiments were performed using 6-10 mice per group.
Data exclusions	No data were excluded from the analyses.
Replication	All experimental findings were successfully reproduced by each atempt.
Randomization	Animals were randomly assigned to mock or pentanoate treatment. Animals of same sex and age were used to control for covariates.
Blinding	Investigators were not blinded.

Reporting for specific materials, systems and methods

We require information from authors about some types of materials, experimental systems and methods used in many studies. Here, indicate whether each material, system or method listed is relevant to your study. If you are not sure if a list item applies to your research, read the appropriate section before selecting a response.

Materials & experimental systems

n/a	Involved in the study	
☐	☒	Antibodies
☒	☐	Eukaryotic cell lines
☒	☐	Palaeontology
☐	☒	Animals and other organisms
☒	☐	Human research participants
☒	☐	Clinical data

Methods

n/a	Involved in the study	
☒	☐	ChIP-seq
☐	☒	Flow cytometry
☒	☐	MRI-based neuroimaging

Antibodies

Antibodies used	The following antibodies were used: anti-CD4 (BioLegend, clone GK1.5, #100402, 1:400), anti-CD4 (BD Biosciences, clone RM4-5, #560468, 1:500), anti-CD19 (Thermo Fisher Scientific, clone eBio1D3, #45-0193-82, 1:400), anti-CD8 (BD Biosciences, clone 53-6.7, #560776, 1:500). anti-IL-17A (Thermo Fisher Scientific, clone eBio17B7, #17-7177-81,1:200), anti-IFN-g (Thermo Fisher Scientific , clone XMG1.2, #17-7311-82, 1:500), anti-phospho-AKTSer437 (#9271, Cell Signaling Technology, 1:1000), anti-phospho-AKTThr308 (#13038, CST, 1:1000), anti-total AKT (#9272, CST, 1:1000), anti-phospho-mTORSer2448 (#5536, CST, 1:1000), anti-total mTOR (#2983, CST, 1:1000), anti-phosphoS6KT389 (#9205, CST, 1:1000), anti-total S6K (#9202, CST, 1:1000), anti-phosphoS6S235/236 (#4858, CST, 1:1000) and anti-total S6 (#2217, CST, 1:1000).
Validation	Validation statements for antibodies are available on the manufacturer's website.

Animals and other organisms

Policy information about studies involving animals; ARRIVE guidelines recommended for reporting animal research

Laboratory animals	Female mice with age between 8-12 weeks were used for this study. WT mice on C57BL/6 background were purchased from Charles River Laboratories. Germ-free mice were reared in sterile plastic isolators and were monitored biweekly for sterility. FIR × tiger reporter mice were bred at the animal facility of the Biomedical Research Center, University of Marburg, Germany.
Wild animals	The study did not involve wild animals.
Field-collected samples	The study did not involve the samples collected from the field.

Ethics oversight	Animal work was approved by Regierungspräsidium Gießen, Germany under project numbers 70/2014 and EX7-2015.

Note that full information on the approval of the study protocol must also be provided in the manuscript.

Flow Cytometry

Plots

Confirm that:

☒ The axis labels state the marker and fluorochrome used (e.g. CD4-FITC).

☒ The axis scales are clearly visible. Include numbers along axes only for bottom left plot of group (a 'group' is an analysis of identical markers).

☒ All plots are contour plots with outliers or pseudocolor plots.

☒ A numerical value for number of cells or percentage (with statistics) is provided.

Methodology

Sample preparation	Lymph node- and spleen-derived cells were collected in RPMI, spun down at 1500 RCF at 4 degrees. T cells were isolated with negative selection and were then washed with ice-cold FACS buffer and stained using the antibodies described above for 20 minutes. For intracellular staining, T cells were restimulated with 50 ng/ml PMA and 750 ng/ml ionomycin in the presence of 10 µg/ml brefeldin A for 4 hours. After fixation and permeabilisation, the cells were stained with antibodies.
Instrument	FACSCalibur cytometer and BD FACSAria III cell sorter (both BD Biosciences)
Software	FlowJo analysis software (TreeStar)
Cell population abundance	The purity of T cells and B cells was determined by using anti-CD4 and anti-CD19 antibodies, respectively (over 96 % purity).
Gating strategy	Gating strategy to sort and analyze T lymphocytes and Bregs is provided in Supplementary Figure 7. In brief, lymphocytes were gated using SSC-A and FSC-A, and the CD4+ T cells were gated using anti-CD4 antibody. For Bregs, anti-CD19 antibody was used.

☒ Tick this box to confirm that a figure exemplifying the gating strategy is provided in the Supplementary Information.
